# Unravelling the impact of aging on the human endothelial lncRNA transcriptome

**DOI:** 10.3389/fgene.2022.1035380

**Published:** 2022-10-21

**Authors:** Maria-Kyriaki Drekolia, Sweta Talyan, Rebeca Cordellini Emídio, Reinier Abraham Boon, Stefan Guenther, Mario Looso, Gabrijela Dumbović, Sofia-Iris Bibli

**Affiliations:** ^1^ Institute for Vascular Signalling, Centre for Molecular Medicine, Goethe University, Frankfurt, Germany; ^2^ Bioinformatics Core Unit, Max Planck Institute for Heart and Lung Research, Bad Nauheim, Germany; ^3^ Institute for Cardiovascular Regeneration, Goethe University, Frankfurt, Germany; ^4^ German Center for Cardiovascular Research (DZHK), partner site Rhein/Main, Frankfurt, Germany

**Keywords:** aging, endothelium, lncRNA, PCAT14, regeneration

## Abstract

The incidence and prevalence of cardiovascular disease is highest among the elderly. There is a need to further understand the mechanisms behind endothelial cell aging in order to achieve vascular rejuvenation and minimize the onset of age-related vascular diseases. Long non-coding RNAs (lncRNAs) have been proposed to regulate numerous processes in the human genome, yet their function in vascular aging and their therapeutic potential remain largely unknown. This is primarily because the majority of studies investigating the impact of aging on lncRNA expression heavily rely on *in vitro* studies based on replicative senescence. Here, using a unique collection of young and aged endothelial cells isolated from native human arteries, we sought to characterize the age-related alterations in lncRNA expression profiles. We were able to detect a total of 4463 lncRNAs expressed in the human endothelium from which ∼17% (798) were altered in advanced age. One of the most affected lncRNAs in aging was the primate-specific, Prostate Cancer Associated Transcript (PCAT) 14. In our follow up analysis, using single molecule RNA FISH, we showed that PCAT14 is relatively abundant, localized almost exclusively in the nucleus of young endothelial cells, and silenced in the aged endothelium. Functionally, our studies proposed that downregulation of PCAT14 alters endothelial cell transcription profile and cell functions including endothelial cell migration, sprouting and inflammatory responses *in vitro*. Taken together, our data highlight that endothelial cell aging correlates with altered expression of lncRNAs, which could impair the endothelial regenerative capacity and enhance inflammatory phenotypes.

## Introduction

Aging is defined as a gradual decline in organism function coupled with marked multi-organ changes in redox balance, DNA integrity, telomere length, mitochondrial function as well as non-coding RNA expression (López-Otín et al., 2013). Recent studies propose that endothelial aging and senescence impact on systemic metabolic changes which result in the development of chronic diseases such as diabetes, obesity and atherosclerosis (Campisi and Di d’Adda Fagagna, 2007; Ting et al., 2021). The impact of vascular aging and vascular decline on the overall youthfulness of tissues is receiving growing attention (Le Couteur and Lakatta, 2010), and interestingly, induction of angiogenesis in aged mice rescued detrimental effects of aging, proposing that preserving endothelial cell regeneration can serve as a rejuvenation strategy (Das et al., 2018). In the same context, the use of senolytics to target endothelial cell senescence was recently proposed as a novel organismal rejuvenation approach (Suda et al., 2021). With the increasing life expectancy, the number of patients with age-related cardiovascular diseases will rise in the near future, leading to an increased healthcare burden. There is a need for new therapies to treat vascular and endothelial cell aging and minimize the onset of age-related diseases.

Currently there are over 16,000 annotated long-noncoding RNAs (lncRNAs) in mammalian genomes (Snyder et al., 2020), which is proportional to the number of annotated protein-coding genes. Over the past two decades, numerous studies demonstrated that lncRNAs can influence gene expression programs, cellular differentiation and organismal development (Mongelli et al., 2019; Rinn and Chang, 2020; Statello et al., 2021). lncRNAs were shown to have important roles in vascular diseases and aging (Bink et al., 2019), regulating endothelial cell cycle control (Tripathi et al., 2013; He et al., 2015; Boon et al., 2016; Hofmann et al., 2019) and inflammatory profiles (Holdt et al., 2010; Cremer et al., 2019). Identification of lncRNAs regulated by aging and studying their downstream molecular targets would potentially unravel novel mechanisms of aging and open new avenues for vascular rejuvenation. Nevertheless, the role of lncRNAs in endothelial cell aging remains largely unknown, in part because most of the studies linking lncRNAs with age-associated diseases and endothelial senescence have been performed in cultured human endothelial cells undergone replicative senescence following multiple duplication cycles or animal models. To address this, here, we mapped the human native endothelial lncRNA expression profiles from young and aged individuals. Collectively, our analysis shows lncRNAs are highly affected by aging in endothelial cells, wherein they may play a causative role in the endothelial dysfunctional phenotypes. Furthermore, we provide a valuable resource for the community to investigate lncRNA expression dynamics in aging of primary endothelial cells.

## Materials and methods

### Materials

Cell culture media and phosphate buffer saline were from Gibco (Invitrogen; Darmstadt, Germany). Fibronectin was from Corning (Corning GmBH, Kaiserslautern, Germany), interleukin (IL)-1β was from Proteintech (Manchester, UK).

### Endothelial cell isolation and culture

Human endothelial cells from mesenteric arteries. Healthy mesenteric arteries from humans of 20±2.3 and 80±3.4 years of age, were isolated as previously described (Bibli et al., 2021).

Human umbilical vein endothelial cells. Cells were isolated and cultured as described (Bibli et al., 2021) and were used (passage up to 3) for the different experiments.

THP-1 cells. THP-1 monocytic cells were obtained from the American Type Culture Collection (LGC Standards) and cultured in RPMI-1640 containing 2 mmol/L glutamine, 10 mmol/L HEPES, 1 mmol/L sodium pyruvate, 4.5 g/L glucose, 1.5 g/L sodium bicarbonate and 10% FBS.

All cells were negative for mycoplasma contamination. Cultured cells were kept in a humidified incubator at 37°C containing 5% CO_2_.

### RNA sequencing

To extract total RNA from endothelial cells (cultured or native), samples were processed with the RNeasy Mini Kit (Qiagen, Hilden, Germany) and subjected to on-column DNase digestion according to the manufacturer’s protocol. RNA and library preparation integrity were verified with LabChip Gx Touch 24 (Perkin Elmer). 4µg of total RNA was used as input for VAHTS Stranded RNA-seq Library preparation following manufacture’s protocol (Vazyme). Sequencing was performed on NextSeq2000 instrument (Illumina) using P3 flowcell with 1 × 72 bp single end setup. The resulting raw reads were assessed for quality, adapter content and duplication rates with FastQC (http://www.bioinformatics.babraham.ac.uk/projects/fastqc). Trimmomatic version 0.39 was employed to trim reads after a quality drop below a mean of Q20 in a window of 5 nucleotides (Bolger et al., 2014). Only reads between 30 and 150 nucleotides were cleared for further analyses. Trimmed and filtered reads were aligned versus the Ensembl human genome version hg38 (Ensembl release 104) using STAR 2.7.9a with the parameter “--outFilterMismatchNoverLmax 0.1” to increase the maximum ratio of mismatches to mapped length to 10% (Dobin et al., 2013). The number of reads aligning to genes was counted with featureCounts 2.0.2 tool from the Subread package (Liao et al., 2014). Only reads mapping at least partially inside exons were admitted and aggregated per gene. Reads overlapping multiple genes or aligning to multiple regions were excluded. Differentially expressed genes were identified using DESeq2 version 1.30.0 (Love et al., 2014). Only genes with a minimum fold change of +- 2 (log2 = +-1), a maximum Benjamini-Hochberg corrected p-value of 0.05, and a minimum combined mean of 5 reads were deemed to be significantly differentially expressed.

### Cell transfection with GapmeRs

For knockdown assays, in three different biological replicates of human umbilical vein endothelial cells were seeded and allowed to reach 40%–50% confluency on the day of transfection. Subsequently, cells were transfected with 50 nmol/L GapmeR targeting PCAT14 or a negative control GapmeR (Qiagen, Paris, France) using Lipofectamine RNAiMAX (Invitrogen, Thermo Fischer Scientific, Dreieich, Germany) according to the manufacturer’s protocol. After 4 hours, the transfection mix was replaced by endothelial growth media and the cells were incubated for additional 72 h. Experiments were repeated three independent times.

### RT-qPCR

Total RNA was extracted using RNeasy kit (QIAGEN, Hilden, Germany) according to manufacturer instructions. Equal amounts of total RNA (1 µg) were reverse transcribed using Superscript III (Invitrogen) according to instructions. Gene expression levels were detected using SYBR Green (Absolute QPCR SYBR Green Mix; Thermo Fisher Scientifc). The relative expression levels of the different genes studied was calculated using the formula 2−∆*C*t [∆*C*t = *C*t (gene) – *C*t (housekeeping gene)] with the 18S RNA as a reference. The primer sequences used were as follows: 18s: forward 5´-CTT​TGG​TCG​CTC​GCT​CCT​C-3´, reverse 5´-CTG​ACC​GGG​TTG​GTT​TTG​AT-3´ and PCAT14: forward 5´-GGC​GCA​GGC​CAC​TCC​ATC​TGG​TG-3´, reverse 5´-CCT​CAA​TGA​CCA​CAC​TGT​AGA​G-3´.

### Single molecule RNA imaging

43 oligonucleotides labeled with Quasar 670 targeting human PCAT14 exon sequence were designed with the Stellaris RNA FISH probe designer and produced by LGC Biosearch Technologies. Human umbilical vein endothelial cells were seeded on glass coverslips coated with fibronectin (25 µg/mL) and gelatin (0.5% w/v, Sigma, Darmstadt, Germany). SmRNA FISH was performed as previously described (Dumbovic et al., 2018; Dumbović et al., 2021). Samples were left to curate at 4°C overnight before proceeding to image acquisition.

### Microscopy and image analysis

Image acquisition was performed using a Nikon ECLIPSE Ti2 widefield microscope with a Nikon Plan Apo λ 100x/1.45-numerical aperture oil objective lens and a Nikon DS-Qi2 camera. Z-stacks of 180 nm step size were acquired. Maximum intensity projections were created using ImageJ/Fiji. FISH spots were quantified manually using ImageJ/Fiji, wherein the brightness and contrast of each channel were adjusted.

### Cell proliferation/confluency

Human umbilical vein endothelial cells were seeded onto 24 well plates (Sarstedt, Germany) at a density of 15,000 cells/well. 16 images/well at magnification × 10 were taken every 4 h using an IncuCyte S3 live-cell analysis system (Essen BioScience, United Kingdom). Cell confluence was measured for up to 4 days. Image processing and analysis were performed using the following parameters: segmentation adjustment 1.8; clean up: hole fill 0 μm^2^; adjust size (pixels): 0; filter: minimum 350 μm^2^ area, no maximum, eccentricity: no minimum, no maximum.

### Wound scratch assay

Human umbilical vein endothelial cells were seeded onto 8 well μ-slides (Ibidi) at a density of 40.000 cells/ well. The cell monolayer was scraped in a straight line with a p200 pipet tip 24 h later and the media was immediately replaced with fresh endothelial growth media. Pictures were taken at 0 and 6 after the wound formation using Axio Observer A1 microscope (Zeiss Oberkochen, Germany) with an AxioCam MRm camera (Zeiss). In order to maintain the same area during the image acquisition marks were created as reference points close to the scratch.

### Spheroid sprouting assay

Spheroids containing 500 cells were generated as described (Pfisterer et al., 2016), and cultured in EBM supplemented with 2.5% FCS and VEGF (30 ng/mL). Phase contrast images were taken after 24 h and sprouting was quantified by measuring the cumulative length of the sprouts originating from an individual spheroid. Imaging was performed using an Axio Observer A1 microscope (Zeiss Oberkochen, Germany) with an AxioCam MRm camera (Zeiss). At least five spheroids per group were analyzed for each cell batch.

### Monocyte adhesion

Confluent human umbilical vein endothelial cells (cultured in µ-Slide 8 well, ibidi GmbH, Germany) were subjected to transfection or stimulation with IL-1β (10 ng/ml, 4 h). Thereafter, THP-1 cells (100.000) were added and left undisturbed for 30 min as previously described (Bibli et al., 2019). Non-adherent cells were removed by washing three times with 1x phosphate buffer saline supplemented with 1mmol/L Ca^2+^ and 1 mmol/L Mg^2+^ and adherent cells were imaged with Axio Observer A1 microscope (Zeiss Oberkochen, Germany) with an AxioCam MRm camera (Zeiss).

### Data analysis and bioinformatics

All graphs were generated using GraphPad Prism 9. Volcano plots were made with EnhancedVolcano package from Bioconductor version 3.15. Hierarchical clustering heatmaps were constructed using RColorBrewer and pheatmap libraries on R software (Clustering method = “average”, clustering distance in rows and columns = “euclidean” or “correlation”).

## Statistics

Data are expressed as mean ± SEM. Statistical evaluation was performed using Student’s t test for unpaired or paired data. Statistical tests are described in the figure legend for each experiment. Values of *p* < 0.05 were considered statistically significant.

## Results

### Endothelial aging impacts on lncRNA expression profiles

**FIGURE 1:**
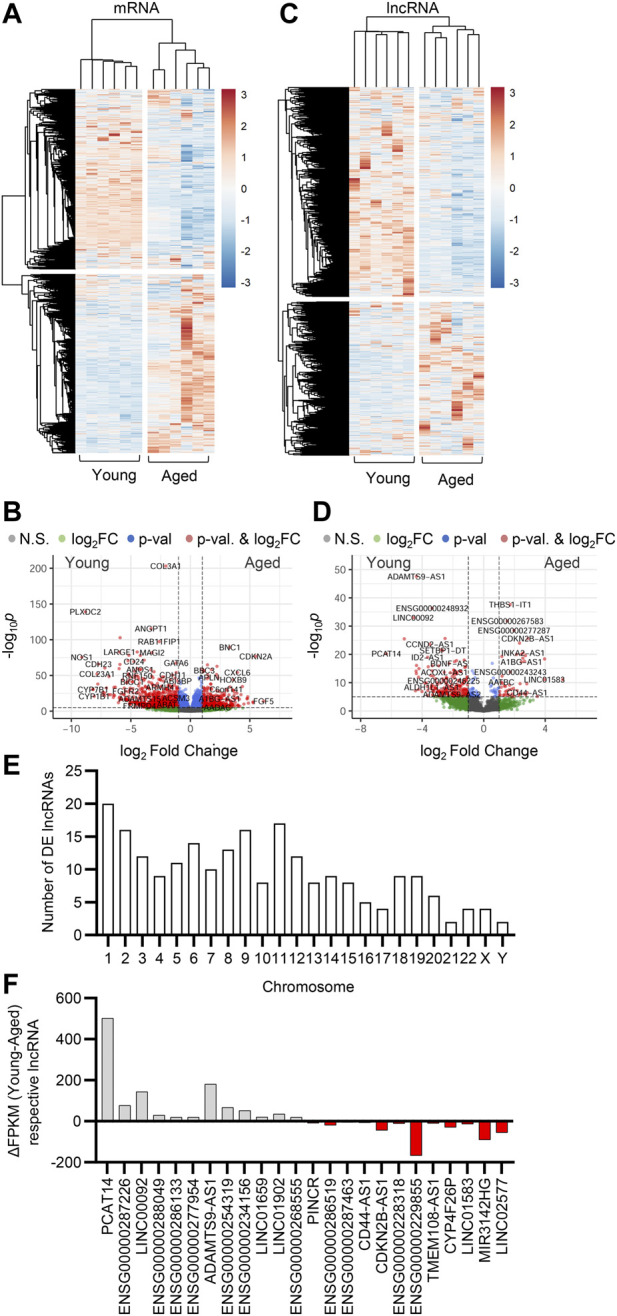
Effect of aging on endothelial cell transcriptome. Bulk RNA-sequencing analysis was performed in human native young and aged arterial endothelial cells (*n*=6 per group, each sample is a pool of 5 different isolates). **(A,C)** Hierarchical clustering heatmap showing differentially expressed mRNAs (A) and lncRNAs (C). **(B,D)** Representative volcano plots showing differentially expressed mRNAs (B) and lncRNAs (D) (total mRNA variables = 15,379, total lncRNA variables = 4,463). **(E)** Chromosome distribution of differentially expressed (DE) lncRNAs detected as in (C) and (D). **(F)** Expression levels presented as ΔFPKM of lncRNA transcripts detected in panels (C) and (D).

It is well established that lncRNA expression is highly cell type specific (Derrien et al. 2012). To study the expression profiles of lncRNAs in the human native endothelium and analyze the impact of aging on the expression of lncRNAs, we performed RNA-sequencing (RNAseq) on native young (average age 20 years) and aged (average age 80 years) arterial endothelial cells. A total of 4,463 lncRNAs were detected (Supplementary Table S1). Hierarchical clustering defined differential expression patterns of both mRNAs and lncRNAs during aging and revealed an age-dependent alteration in the transcriptome of the human endothelium (Figure 1A,C). Transcripts encoding proteins related to endothelial homeostasis and integrity (i.e., PLXDC2, NOS1, ANGPT1, FGFR2, and CDH23) as well as metabolic related transcripts (i.e., LARGE1, CYP7B1 and CYP1B1) were downregulated during aging, whilst genes associated with disease, apoptosis and inflammation were upregulated (i.e., BBC3, CXCL6, CDKN2A, and BNC1) (Figure 1B). We observed a significant transcriptional reprogramming of lncRNAs, with 601 lncRNAs being downregulated ( ≤ -1 log_2_ fold change, *p*<0.05) and 197 lncRNAs being upregulated ( ≥ 1 log_2_ fold change, *p*<0.05) in the aged human endothelium (Figure 1D). To determine whether the differentially expressed (DE) lncRNAs originate from specific chromosomes and chromosome regions, which would therefore reflect epigenetic landscape changes known to occur in upon aging (Ding et al., 2020), we analyzed the distribution of affected lncRNAs in the genome. The differentially expressed lncRNAs were distributed among the chromosomes, with the majority of them positioned on chr1, chr2, chr6, chr8, chr9, and chr11 (Figure 1E). lncRNAs whose expression was most affected in the aged human endothelium compared to endothelial cells isolated from young arteries were PCAT14, LINC00092, and ADAMTS9-AS1 (Figure 1F). Overall, our analysis shows that expression of lncRNAs is significantly affected by aging in primary endothelial cells, with many of them being involved in the regulation of endothelial cell function.

**FIGURE 2:**
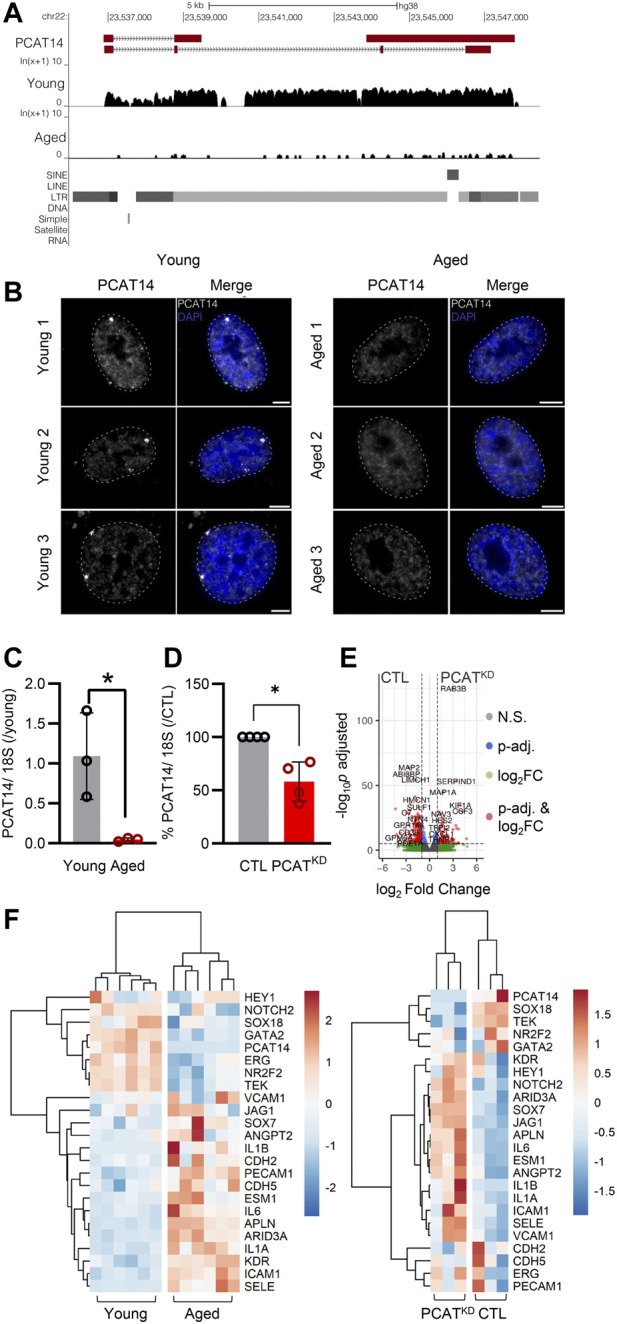
PCAT14 expression, localization and impact on endothelial cell transcriptome. **(A)** UCSC Genome Browser showing the PCAT14 locus (hg38) and the poly (A)+ RNA-seq tracks from young and aged human native endothelial cells. **(B)** Maximum intensity projections of representative images of PCAT14 smRNA FISH on young and aged human native endothelial cells. Exon, gray; nucleus, blue, outlined with a dashed circle. Scale bar = 5 μm. RNA FISH was performed on 7 young and 4 aged samples. **(C)** Relative lncRNA levels of PCAT14 in young and aged native isolated arterial endothelial cells (*n*=3 per group, *p<0.05, Student’s t-test). **(D)** Relative PCAT14 expression normalized to CTL in Human umbilical vein endothelial cells (HUVEC, Passage 3) were transfected with GapmeR (50 nmol/L, 72 h) targeting PCAT14 (PCAT^KD^) or a respective control (CTL). **(E)** Representative volcano plots showing differentially expressed transcripts (total variables = 22,059) in samples as in Figure 1D. **(F)** Hierarchical clustering heatmap showing the differentially expressed levels of PCAT14, endothelial and inflammatory markers in human native young and aged arterial endothelial cells (*n*=6 per group, each sample is a pool of 5 different isolations) and in human umbilical vein endothelial cells CTL or PCAT^KD^ as in (D). N.S: Not Significant; log_2_FC: log_2_ (Fold Change); *p*-val: *p*-value.

### PCAT14: the major endothelial age-dependent downregulated lncRNA

**FIGURE 3:**
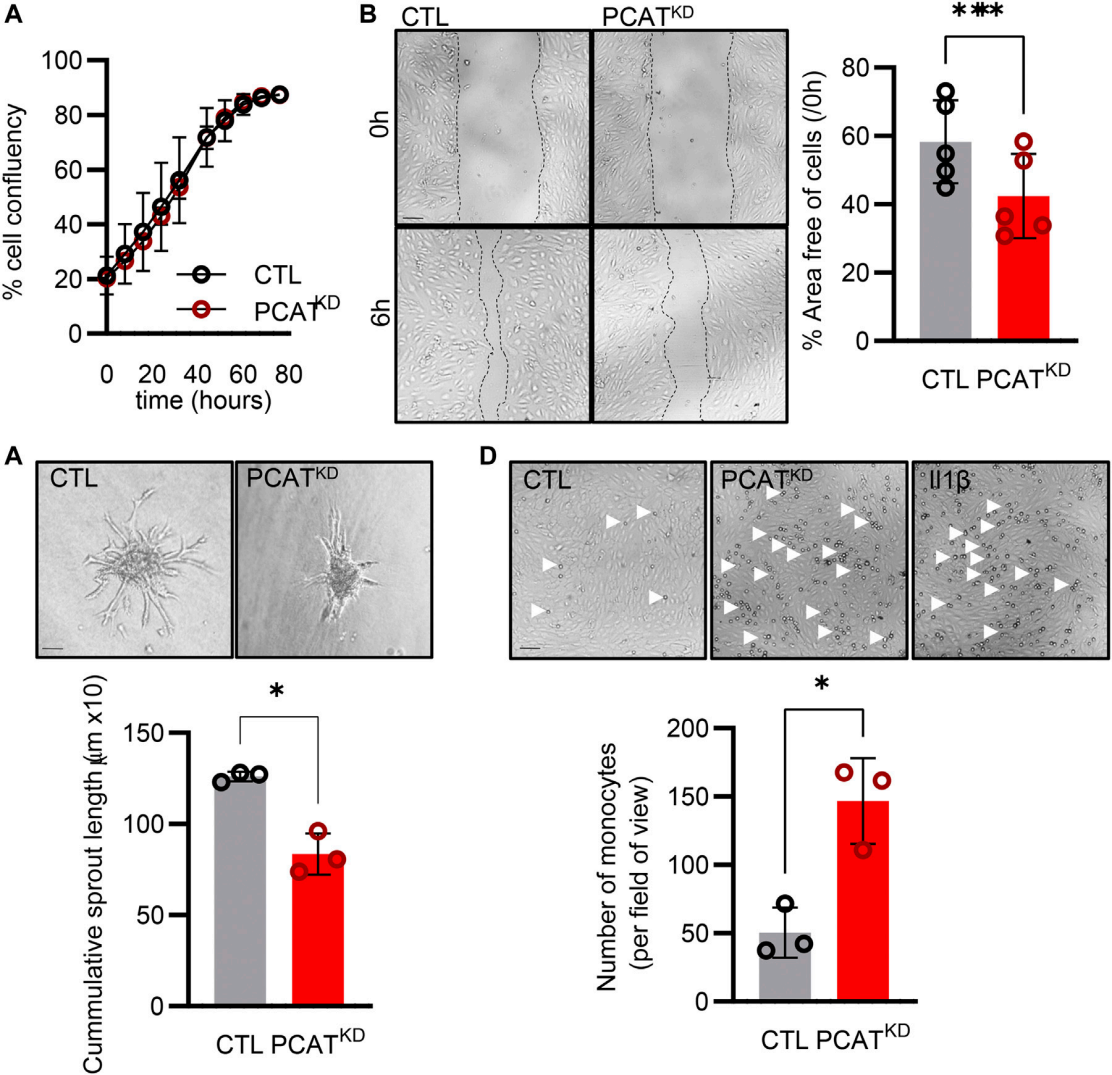
Effect of PCAT14 deletion on endothelial angiogenic responses and activation. Human umbilical vein endothelial cells (HUVEC, Passage 3) were transfected with a GapmeR (50 nmol/L, 72 h) targeting PCAT14 (PCAT^KD^) or a respective control (CTL). **(A)** Percentage of endothelial cell confluence for up to 48 h. **(B)** Representative brightfield images and quantification following a wound scratch assay at 0 and 6 hours. **(C)** Representative brightfield images and quantification of endothelial cell sprouts on a 3D spheroid assay. **(D)** Representative brightfield images and quantification of THP-1 monocytes adhered to endothelial cells. As a positive control, endothelial cells treated with Interleukin 1β (Il-1β, 10 ng/mL, 4 h). *n*=3-6 biological replicates per group, experiments performed at least 2 times. Scale bar = 500 μm, *p<0.05, **p<0.01, Student’s t-test paired, N.S: Not Significant; log_2_FC: log_2_ (Fold Change); *p*-adj: *p*-value adjusted.

Given that the lncRNA PCAT14 was among the most highly abundant expressed lncRNAs in the native human endothelium shown to be reduced in the aged endothelial cells, we focused our further studies on PCAT14. A detailed analysis of PCAT14 locus revealed interesting and distinguishing features: i) PCAT14 is not in a vicinity of any protein coding gene, the nearest one being IGLL1 (Immunoglobulin lambda-like polypeptide 1) located approximately 25 kb downstream of the PCAT14 locus and ii) PCAT14 locus overlaps with a human specific endogenous retrovirus K element (HERVK), therefore making it a primate-specific, transposon-derived lncRNA (Figure 2A). We used single molecule RNA imaging (smRNA FISH) targeting PCAT14 exons to analyze the distribution of PCAT14 lncRNA in human native endothelial cells isolated from individuals of different age. Our analysis shows that PCAT14 is almost exclusively a nuclear lncRNA, with most clear signal forming bright punctuate foci in young endothelial cells (Figure 2B). These foci could likely be accumulations of nascent PCAT14 transcript *in cis*. Interestingly, these foci are most frequently localized at the nuclear periphery or lamina, which are regions known to consist of heterochromatin. By quantitative RT-PCR (RT-qPCR) we validated its relatively high expression in young endothelial cells and significant reduction in expression in aged endothelial cells (Figure 2C). To address the role of PCAT14 lncRNA in endothelial cells, we performed RNAseq on human umbilical vein endothelial cells (HUVEC) transfected with a GapmeR against PCAT14 (PCAT^KD^) or a control GapmeR (CTL). By RT-qPCR we show that PCAT14 GapmeRs successfully downregulated its expression by approximately 60% (Figure 3A). Knockdown of PCAT14 was accompanied by 2,497 significantly dysregulated genes compared to endothelial cells treated with the respective control (Fold Change>2.0 or <−2.0 and p<0.05, Figure 2E). A side-by-side comparison of transcripts involved in endothelial cell identity, i.e. ERG, KDR, CDH5, ANGPT2, PECAM1, between young and aged endothelium showed that aging affects the expression of genes involved in endothelial cell identity. In contrast, loss of PCAT14 does not affect the key endothelial cell markers. However, similar to the human native aged endothelium, knockdown of PCAT14 increased the expression of inflammatory genes, i.e., ICAM1 and SELE. In addition, PCAT14 downregulation increased the expression of genes relevant to endothelial cell stalk formation, i.e., HEY1, JAG1, and ESM1 (Figure 2F).

### PCAT14 regulates key endothelial cell functions

Knockdown of PCAT14 did not affect the endothelial proliferative capacity (Figure 3A). However, diminished PCAT14 expression reduced the endothelial cell migratory ability approximately by 30% (Figure 3B) and reduced the sprouting capacity by approximately 35% (Figure 3C). These data indicate that PCAT14 preserves endothelial regenerative and angiogenic properties. Finally, we investigated the pro-inflammatory phenotypes of the PCAT^KD^ endothelial cells through an endothelial cell-monocyte adhesion assay. Human monocytes (THP-1) exhibited increased capacity to adhere to endothelial cells lacking PCAT14, to a similar extent as endothelial cells stimulated prior with interleukin 1 beta (Il-1β) for 4 h, indicating that PCAT14 impacts on endothelial cell activation (Figure 3D).

## Discussion

Our data highlight that aging alters the endothelial cell lncRNA expression profiles. We identified 798 lncRNAs being significantly altered in the aged human native endothelium. Focusing on one of the most endothelial abundant and simultaneously age sensitive lncRNA, PCAT14, it was possible to link its expression with the preservation of endothelial cell migration, sprouting as well as anti-inflammatory potential *in vitro*.

Advanced aging is an independent risk factor related with the development of endothelial dysfunction even in the absence of a clinical disease. With advanced age the endothelium is characterized by increased inflammation, oxidative stress and senescence (Campisi and Di d’Adda Fagagna, 2007). Endothelial cell senescence is coupled with genomic instability, telomere attrition and DNA damage which are able to trigger the p53/p21 pathway and eventually lead to cell cycle arrest (van Deursen, 2014; Donato et al., 2015). Notably, the elimination of senescent cells (senolysis) was recently reported to improve normal and pathological changes associated with aging in mice (Baker et al., 2016; Xu et al., 2018) and the recent development of endothelial cell-specific senolytic targeting seno-antigens could be a promising strategy for new senolytic therapies to diminish pathological phenotypes associated with aging (Suda et al., 2021).

Although extensive work has been done to identify and characterize lncRNAs in multiple diseases (He et al., 2018), including cellular senescence (Bink et al., 2019), only a few have been examined for their potential actions in the endothelial aging and age-associated vascular diseases. Among those linked to vascular aging, MALAT1, H19, and GFADS5 have been shown to impact endothelial function and induce aging by modulating the expression of the cell cycle regulators (Michalik et al., 2014), or regulate the endothelial epigenome and its angiogenic responses (Hofmann et al., 2019; Jiang and Ning, 2020). The lncRNA Mirial was recently shown to be downregulated in the aged murine endothelium and impact on endothelial metabolic and cellular functions (Kohnle et al., 2021). The lncRNA ANRIL seems to be increased in advanced endothelial aging both in murine models (Tsai et al., 2010) and patients with coronary artery disease, atherosclerosis and type 2 diabetes. Its actions have been attributed to its ability to bind either on p300 and affect VEGF expression or on polycomb repressive complex-2 (PRC2) complex (Thomas et al., 2017) and disturb its binding to the p15^INK4B^ locus (Kotake et al., 2011). The lncRNA HOTAIR is considered to affect also the PRC2 and the lysine-specific histone demethylase-1 complexes and affect the levels of H3K27me3 and H3K4me2 (Tsai et al., 2010), inducing atherosclerotic events during aging (Pierce and Feinberg, 2020). The lncRNAs Meg3 and lincRNA-p21 affect p53 expression and are linked to vascular aging (Huarte et al., 2010; Shihabudeen Haider Ali et al., 2019). The current literature links the aforementioned lncRNAs with endothelial cell function and development of age-related characteristics or altered endothelial cell signaling responsible for the development of age-related phenotypes. In our datasets we were able to validate that in the native human aged endothelium the lncRNA H19 was significantly downregulated and ANRIL was significantly upregulated. MALAT1, Meg3, GFADS5, and HOTAIR were indeed expressed in the human native endothelium, however, their expression profiles seem to be age independent. This might be attributed to the fact that studies investigating these lncRNAs rely on the *in vitro* induced endothelial replicative senescence is mostly related to the telomere attrition and it can only partially mimic the *in vivo* complex phenomena taking place in the progression of cellular aging. An alternative explanation would be that the current literature investigates the expression profiles of the aforementioned lncRNAs in the presence of additional co-morbidities i.e. atherosclerosis, diabetes or carotid artery diseases, which sometimes precede endothelial senescence.

lncRNAs identified as being the most downregulated in the human native aged endothelium have been described in the context of cancer. In particular, ADAMTS9-AS1 is highly expressed in hepatocellular carcinoma cells and it exacerbates cell proliferation, migration and invasion through the PI3K/AKT/mTOR pathways (Zhang et al., 2020). LINC00092 affects the glycolysis levels and modifies the Warburg effects in ovarian cancer progression and metastasis (Zhao et al., 2017). Given that endothelial cells heavily rely on glycolysis, further studies investigating LINKC000092 in the context of endothelial rejuvenation, would be promising. The lncRNA CCND2-AS1 has been identified in papillary thyroid carcinoma, breast cancer and cervical cancer and promotes proliferation, migration and invasion (Chen et al., 2018). In contrast to the most down-regulated lncRNAs which are linked to cell proliferation, migration and invasion, the most upregulated lncRNAs were linked to senescence. In particular, THBS1-IT1, highly expressed in the centenarian population, has been linked to decrease of p16, p21 and the activity of the senescent related beta-galactosidase (Jiang et al., 2021).

We focused our further studies on PCAT14 due to its high expression in native young endothelium which was not detected in the aged endothelium. PCAT14 is mostly expressed in the prostate and the testis and less in the thyroid, lung and ovary. At the same time, PCAT14 has been characterized as a novel prostate cancer and lineage-specific lncRNA (Shukla et al., 2016; Yan et al., 2021). In the native human endothelium, PCAT14 was one of the most abundant age-sensitive lncRNAs. An interesting observation is that PCAT14 originates from HERVK, a primate-specific endogenous retrovirus. HERVK family has been of great interest due to being the most recently acquired HERV from which multiple insertions have retained protein-coding potential (Subramanian et al., 2011). HERVK was found to be transcriptionally active during normal human embryogenesis from the 8-cell stage until the stem cell derivation (Grow et al., 2015). Most HERVKs are transcriptionally silenced in healthy adult cells and reactivated in certain pathological contexts, such as cancer or HIV infection (Herbst et al., 1996; Muster et al., 2003; Contreras-Galindo et al., 2008; Bhardwaj et al., 2014).

In our smRNA FISH analysis on native endothelial cells from young and aged individuals, we observed PCAT14 as almost exclusively nuclear lncRNA in native young endothelium. The distribution of PCAT14 points towards it functioning *in cis*, while the extensive silencing of PCAT14 locus upon aging suggests epigenetic reprograming of the locus upon aging. Important to note, although we detect PCAT14 expressed in young endothelium, it is normally silenced in differentiated tissues. The high expression of PCAT14 in young endothelial cells may point towards potential roles of HERVK in endothelial regenerative capacity. Further studies will be needed to determine the exact molecular mechanism of PCAT14 lncRNA and the regulation of the locus.

Interestingly, while knockdown of PCAT14 *in vitro* did not affect endothelial cell identity, it induced the expression of transcripts linked to endothelial cell stalk identity as well as increased the expression of adhesion molecules, which are responsible for the pro-inflammatory phenotypes of the endothelium. In addition, ARID3A levels which are increased in patients with pulmonary artery hypertension (PAH) (Reyes-Palomares et al., 2020), were also found increased in cells with a diminished expression of PCAT14. ARID3A is related to endothelial activation and has been shown to regulate p53 functions (Lestari et al., 2012). Given such interactions, we investigated whether PCAT14 would have potential impact on endothelial cell migration, sprouting and inflammatory responses. Indeed, by knocking down PCAT14 in young endothelial cells during proliferation we observed diminished migratory capacity, reduced sprouting as well as increased activation.

Taken together, our data highlight that aging can strongly affect lncRNA expression profiles, which may potentially affect endothelial fitness. Downregulation of one such age-sensitive lncRNA, PCAT14, affects major endothelial functions *in vitro*. Therefore, going forward, it will be important to investigate the individual and/or combined roles of lncRNA expression alterations and mechanisms thereof on endothelial cell functions.

## Data Availability

The data presented in the study are deposited in the GEO, under the accession number GSE214476 (https://www.ncbi.nlm.nih.gov/geo/query/acc.cgi?acc=GSE214476) and GSE214478 (https://www.ncbi.nlm.nih.gov/geo/query/acc.cgi?acc=GSE214478).
